# Supplementary material for: A high-resolution mRNA expression time course of embryonic development in zebrafish
Source: eLife. 2017 Nov 16;6:e30860. doi: 10.7554/eLife.30860 (PMC5690287; doi:10.7554/eLife.30860)
Supplement: Supplementary file 6. [file elife-30860-supp6.zip › biolayout-clusters-files/Cluster222-genes.html]

Cluster222


# Cluster222: Genes

| Ensembl ID | Gene Name | Chr | Start | End | Biotype |
| --- | --- | --- | --- | --- | --- |
| ENSDARG00000068934 | cyp1b1 | 13 | 41885315 | 41892978 | protein\_coding |
| ENSDARG00000030999 | mybl1 | 24 | 23646665 | 23672609 | protein\_coding |
| ENSDARG00000036816 | pou2f2a.1 | 19 | 6294649 | 6384068 | protein\_coding |
| ENSDARG00000102113 | si:ch211-193l2.7 | 11 | 41449985 | 41453173 | protein\_coding |
| ENSDARG00000105429 | si:ch211-193l2.8 | 11 | 41441134 | 41442484 | lincRNA |
| ENSDARG00000010207 | smad3b | 18 | 19783808 | 19817143 | protein\_coding |
